# Color and cellular selectivity of retinal ganglion cell subtypes through frequency modulation of electrical stimulation

**DOI:** 10.1038/s41598-021-84437-w

**Published:** 2021-03-04

**Authors:** Javad Paknahad, Kyle Loizos, Lan Yue, Mark S. Humayun, Gianluca Lazzi

**Affiliations:** 1 Department of Electrical Engineering, University of Southern California, Los Angeles, CA USA; 2 The Institute for Technology and Medical Systems (ITEMS), Keck School of Medicine, University of Southern California, Los Angeles, CA USA; 3 Roski Eye Institute, University of Southern California, Los Angeles, CA USA; 4 Departments of Ophthalmology and Biomedical Engineering, University of Southern California, Los Angeles, CA USA

**Keywords:** Computational models, Retinal diseases

## Abstract

Epiretinal prostheses aim at electrically stimulating the inner most surviving retinal cells—retinal ganglion cells (RGCs)—to restore partial sight to the blind. Recent tests in patients with epiretinal implants have revealed that electrical stimulation of the retina results in the percept of color of the elicited phosphenes, which depends on the frequency of stimulation. This paper presents computational results that are predictive of this finding and further support our understanding of the mechanisms of color encoding in electrical stimulation of retina, which could prove pivotal for the design of advanced retinal prosthetics that elicit both percept and color. This provides, for the first time, a directly applicable “amplitude-frequency” stimulation strategy to “encode color” in future retinal prosthetics through a predictive computational tool to selectively target small bistratified cells, which have been shown to contribute to “blue-yellow” color opponency in the retinal circuitry. The presented results are validated with experimental data reported in the literature and correlated with findings in blind patients with a retinal prosthetic implant collected by our group.

## Introduction

Retinal and cortical visual prostheses have been developed to restore partial sight to the patients who have been blinded for decades by neurodegenerative diseases, such as retinitis pigmentosa (RP), age-related macular degeneration (AMD), and Primary Open-Angle Glaucoma (POAG). Restoration in vision lost has been generally attempted by either stimulating the surviving neurons in the degenerated retina to elicit visual percepts or bypassing the visual pathway and directly stimulating the visual cortex. These approaches have proven effective and led to the development of several visual prosthetic systems^1–6^.

The target of electrical stimulation in epiretinal prostheses is the innermost layer of the retina—the population of retinal ganglion cells (RGCs)—which remain mostly intact in the early stages of degeneration. Research has been conducted towards improving the efficacy and safety of such devices using computational and experimental methods^7–23^. While these devices have shown to be effective at restoring some limited form of sight, several challenges still need to be addressed. A critical issue with current epiretinal prosthetic systems, for example, is the limited ability to focally activate a population of RGCs. Reports from clinical studies have revealed that axonal activation of RGCs can result in elongated phosphenes^7^. Direct and indirect electrical stimulation of RGCs have been attempted using long and short pulse durations to achieve more focalized response from a population of RGCs^10–15^. However, percept fading and desensitization with indirect stimulation, and high required current amplitude with direct stimulation of RGCs remained a challenge^16^.

Further understanding of how different subtypes of RGCs respond to electrical stimulation, and the mechanisms underlying the preferential activation of each cell type, could significantly improve the efficacy of retinal prostheses. A number of studies have focused on RGCs excitability to high frequency electrical stimulation (up to 300 Hz)^8,17–20^. Further, there have been attempts towards preferentially targeting ON and OFF RGCs at very high stimulation frequency (> 2 kHz)^21–23^. Despite these successes, to the best of our knowledge there has been no specific work on analyzing the responsiveness of classified RGCs subtypes to high frequency of stimulation. Prior studies have been mostly limited to the response to light stimuli of ON and OFF RGCs, or one morphological RGC type to electrical stimulation. However, there are subtypes of RGCs within each group (ON, OFF, and ON–OFF) that are characterized by physiological and morphological differences^24–27^. These classified RGCs carry specific types of visual information, such as color and contrast, features which may therefore be possible to leverage in a prosthetic through selective stimulation. For example, previous studies have shown the contribution of small bistratified ganglion cells to “blue-yellow” color opponency in the retinal circuitry^28–31^.

Recent clinical studies of patients with retinal prostheses have shown that electrical stimulation can result in some variation of color perception^32–36^. Specifically, these experiments revealed that color percept is dependent upon stimulation parameters such as frequency of stimulation^32^. These findings suggest the possibility of encoding color in retinal prostheses. Significant loss of spatial visual information in degenerate retina with respect to normal vision is inevitable; indisputably, the addition of color vision would represent a tremendous improvement to the efficacy of current devices.

In this work, we developed biophysically and morphologically detailed models of D1-bistratified and A2-monostratified RGCs and validated their response with experimentally recorded signals^37^. We utilized our combined Admittance method (AM)/NEURON multiscale computational method to determine whether different RGCs exhibit different responses as a function of the stimulation frequency (up to 200 Hz). We found that D1-bistratified cells are better able to follow high stimulus frequency compared to A2-monostratified cells. Our computational platform helps gain further insights into the underlying mechanisms affecting the differential excitability of RGCs at high frequency. This differential response of RGCs with the proper current amplitude modulation can help identify the mechanisms linked to preferential activation of RGCs, and different color percepts observed in clinical studies.

## Results

### Extracellular stimulation: frequency response of RGCs

**Figure 1:**
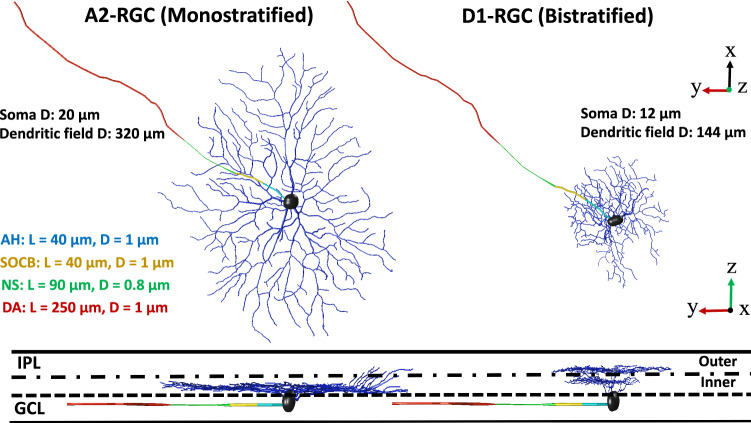
A2 and D1 realistic morphologies as implemented and coded in our multiscale Admittance Method/NEURON computational platform^61–73^. Left: A2-monostratified RGC ramified in the inner part of inner plexiform layer and has a larger soma and dendritic field diameters. Right: D1-bistratified, their dendrites are placed in both inner and outer part of the inner plexiform layer and this cell has relatively smaller soma and dendritic field diameters. GCL: ganglion cell layer; IPL: inner plexiform layer; AH: axon hillock; SOCB: sodium channel band; NS: narrow segment; DA: distal axon; L: length of each band; D: diameter. The morphology of RGCs was extracted from the NeuroMorpho dataset^75–77^.

**Figure 2:**
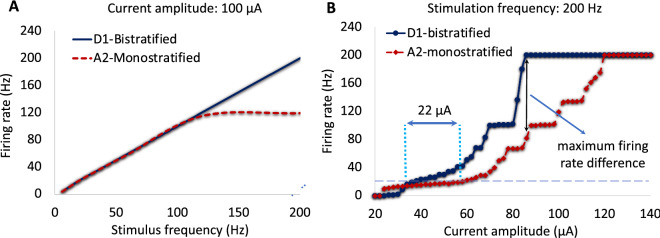
Responsiveness of RGCs at high stimulation frequency. (**A**) Computational results show the difference in response between A2 and D1 retinal ganglion cells at high frequency. (**B**) Firing rate as a function of pulse amplitude for both A2 and D1 cells at 200 Hz stimulus frequency. Data show the effects of stimulus amplitude on the responsiveness of both cell types. Slower rate of changes in firing rates of A2-RGC with increasing amplitude is shown which indicates less excitability of this RGC subtype at high frequency. The greatest difference in rate of firing between A2 and D1 cells is observed at the point where D1 cell begins firing at its maximum rate of 200 Hz. The difference in the computationally determined frequency response can potentially help identifying the mechanism to selectively target RGCs.

The morphology of the two developed RGCs, D1-bistratified versus A2-monostratified, and the levels of stratification in the inner plexiform layer of the retina are depicted in Fig. 1. The stimulating electrode of diameter 200 μm is placed on the top-center of the bulk retina tissue and is positioned 50 µm from the cell bodies of computational models of the RGCs. We applied symmetric charge-balanced electrical stimulation waveforms to characterize RGCs responsiveness as a function of stimulus frequency. We compared the responses of D1-bistratified versus A2-monostratified RGCs to alterations in stimulation frequency. Figure 2A shows the firing rates of both A2 and D1 cells as a function of stimulation frequency at 100 µA current amplitude. As shown in the figure, the firing rate of the D1 cell is greater compared to the A2 cell at high frequency, and the spiking rate observed in the A2-monostratified cell cannot follow the stimulus pulses with a similar rate. However, each stimulus pulse results in spiking of the D1-RGC. The importance of this finding lies in the potential to exploit the differential RGCs response in retinal prosthetic systems by varying stimulation frequency to controllably induce different percepts such as color.

### Current modulation at high frequency

Figure 2B represents the rate of spikes as a function of current amplitude ranging from 20 to 140 µA at 200 Hz. As shown, firing rate increases with increasing stimulus strength up to 100% response probability (200 Hz firing rate) for each cell. However, slower rate of increase in firing rate is observed for A2 cells compared to D1 cells. The differential excitation rates of the RGCs increases with increasing the pulse amplitude. D1-RGC reaches its maximum firing rate at 86 µA, as noted in Fig. 2B. This further indicates that the D1 cell is more responsive at high stimulus frequency over a range of current amplitudes. Figure 2B also shows that with a proper choice of current amplitude, D1 cells can be selectively activated at 200 Hz. The typical stimulus frequency used in epiretinal prosthetic systems is 20 Hz, and RGCs are capable of firing at the same rate. One of the hypotheses is that we can control the cells’ firing rate to remain at 20 Hz by tuning the current amplitude at 200 Hz stimulus frequency, and therefore increase the likelihood for selective activation of D1 cells. For example, the intersections of the horizontal dashed line and the response curves in Fig. 2B represents the current amplitude difference between the cells (~ 22 µA) required to achieve 20 Hz firing rate. While the current amplitude to reach 20 Hz spiking rate for the A2-cell is 58 µA, this current is almost 22 µA smaller for the D1-cell, offering a current window for selective activation of this cell.

Many studies have investigated RGCs response to a single stimulus pulse^12,14,38,39^. However, the stimulation threshold differences among RGCs are small at low frequencies, which makes the potential for preferential activation of RGCs challenging^39^. For instance, the difference in the stimulus thresholds of the A2 and D1 RGCs in response to a single stimulation pulse is only 1.6 µA (The A2 cell threshold: 27.3 µA; the D1 cell threshold: 25.7 µA), reducing the current window for targeting the D1 cell. Therefore, this control of excitability of cells over a range of stimulation frequencies is effective for selective activation of RGCs and is attainable with proper selection of stimulus frequency and current amplitude. Under the assumption that small bistratified retinal ganglion cells play a significant role in the percept of the blue color, our findings correlate well with early experimental results in patients with epiretinal implants^32–36^, perceiving blue as the dominant color in their visual percept at high frequency of stimulation as discussed in the section discussing our results in a patient.

### Time course response at high frequency

**Figure 3:**
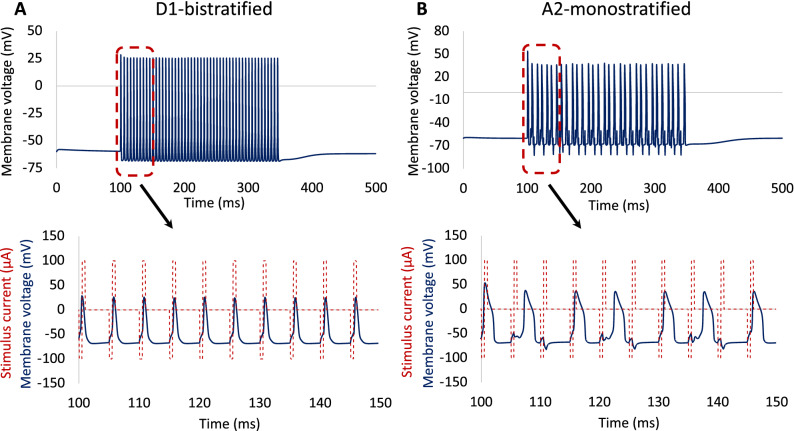
Time course of RGCs response. Membrane potential as a function of time at stimulation frequency of 200 Hz and 100 µA current amplitude: (**A**) D1-bistratified. (**B**) A2-monostratified. D1 cells can better sustain repetitive spikes at high frequency of stimulation compared to A2 cells.

To better understand the physiological differences between these two RGCs, the time course of the response was compared at 200 Hz using a symmetrical biphasic pulse train with a stimulation duration of 250 ms, as indicated in Fig. 3. The results demonstrate that the spiking rate observed in the A2-monostratified cell cannot follow the stimuli pulses at a similar rate. In contrast, each stimulus pulse results in depolarization events in the D1-bistratified cell. There are electrophysiological properties that are different between these two RGC subtypes. It can be clearly seen that the spike width of the D1-RGC is shorter than that of the A2-RGC. In addition, there is a spike latency in the A2 cell response to some of the stimulus pulses, offering an additional reason for lower responsiveness of this cell at high stimulus frequency. This agrees with experiments on RGCs, showing that retinal ganglion cells with longer spike latency cannot sustain repetitive firing at high frequency^18^. There are also morphological factors that can influence their response to high rate of stimulus pulses.

### Sensitivity and statistical analysis of RGCs morphology

We further investigated the effects of morphological changes on response of RGCs to high stimulation frequency. We performed a parametric analysis for a larger population of RGCs taking into account morphological variations within a single RGC type. We separately altered the diameters of the soma and axon within one standard deviation of the mean for both cells based upon the quantitative data available from the literature^25,26^. Then, the weighted average firing rates (WAFR) of each cell at two stimulation frequencies of 120 Hz and 200 Hz were computed (Table 1). We focused our analysis on RGCs response at high frequency because of our interests in excitability of cells at a high rate of stimulation.

**Table 1:** RGCs quantitative data and responses at 120 Hz and 200 Hz.

Measure	RGC types							
	A2-monostratified				D1-bistratified			
	Axon diameter (μm) mean ± SD		Soma diameter (μm) mean ± SD		Axon diameter (μm) mean ± SD		Soma diameter (μm) mean ± SD	
Morphological data	1 ± 0.2		23 ± 4		0.9 ± 0.1		14 ± 3	
SF (Hz)	120	200	120	200	120	200	120	200
WAFR (Hz)	116	121	114.9	113.7	120	185.2	119.5	152.5

In case of modulations in the axon diameter, the soma diameters of the A2 and D1 RGCs were set to 20 µm and 12 µm, respectively. The axon diameters of A2 and D1 cells were fixed to 1 µm and 0.9 µm, respectively for the soma diameter modulations analysis.

*SF* Stimulus frequency, *WAFR* Weighted average firing rate.

**Figure 4:**
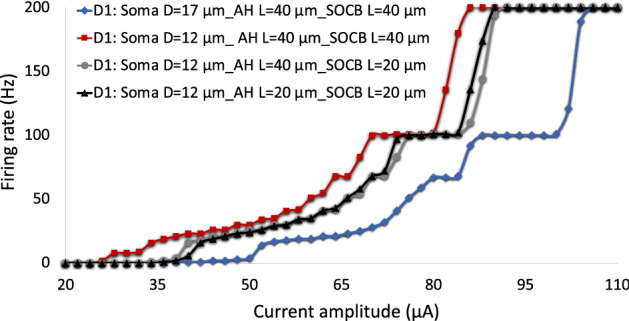
The impacts of the AH and SOCB length on D1-RGCs sensitivity to high frequency electrical stimulation relative to the soma diameter. Firing rate is plotted as a function of modulations in current amplitude at 200 Hz. Analysis of firing rate with the single variation of morphological parameters: soma diameter, SOCB length, and AH and SOCB lengths. Results show that while reduction in the length of the SOCB and AH decreases the responsiveness of D1 cells to high stimulus frequency, the influence of increase in the soma diameter (from 12 to 17 µm) on the reduced sensitivity of the cell to high stimulus frequency is more pronounced.

While spiking activity in both cells follows the monotonous stimulus pulse at 120 Hz, the overall WAFR of D1-bistratified cells is greater than A2-monostratified cells at 200 Hz considering changes in both soma and axon diameters. Both soma and axon diameters influence RGCs firing rates, however the impact of soma diameter is more pronounced at high frequency. We also considered the effects of the sodium channel band (SOCB) and axon hillock (AH) length modulations on sensitivity of RGCs to high frequency electrical stimulation. Recent studies have shown that cells with smaller soma size may have in average smaller SOCB and AH length^39,40^. Therefore, we decreased the length of the SOCB and AH in D1 cells with 12 µm soma diameter (from 40 to 20 µm) and compared the sensitivity of D1 cells to high stimulus frequency with D1 cells having the soma size of 17 µm as shown in Fig. 4. Although the reduced length of the SOCB has lowered the stimulus threshold, we observed that the contribution of soma size alterations to the sensitivity of RGCs to high stimulus frequency remains superior.

**Figure 5:**
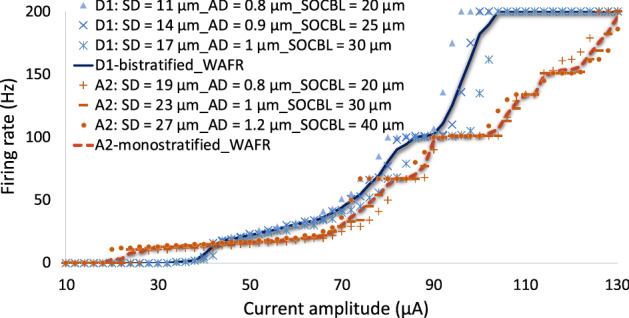
Response (firing rate) of A2 and D1 RGCs to electrical stimulation at 200 Hz with modulations in morphometric parameters. The soma diameter (SD), axon diameter (AD), and SOCB length (SOCBL) alterations of the two cells within one standard deviation of the mean have been investigated. A2 RGCs: SD = 23 ± 4 μm; AD = 1 ± 0.2 μm; SOCBL = 30 ± 10 μm. D1 RGCs: SD = 14 ± 3 μm; AD = 0.9 ± 0.1 μm; SOCBL = 25 ± 5 μm. The weighted average firing rate (WAFR) of the cells indicates the higher excitability of D1 cells at high frequency with relatively smaller SD, AD, and SOCBL.

Given the positive correlations of the soma diameter, axon diameter, and axon initial segment (AIS) lengths, we investigated the firing rates of these RGCs as a function of amplitude modulations at 200 Hz. We incorporated modulations in soma diameter, axon diameter, and SOCB length within one standard deviation of the mean for both cells (Fig. 5)^25–27,39,40^. The WAFR of D1 cells remained greater relative to A2 cells, suggesting the strong contribution of the soma diameter to the responsiveness of RGCs at high frequency. We found a slower rate of change in the number of spikes of A2 cells compared to D1 cells, indicating the difference in the kinetics and densities of ionic channels across RGCs may also influence the rate of spikes at high stimulation frequency. The A2 cell response further shows lower sensitivity to modulations in morphological parameters than that of the D1 cell at high firing rates (Fig. 5). In the next section, we validate our findings with experiments on epiretinal electrical stimulation of A2-type RGCs^8^, showing that small cells can better maintain their response at high stimulus frequency compared to large cells.

### Verification of computational results with in-vitro experiments

To consolidate our observations on the impacts of morphological structure, we reproduced the experimental results on the responsiveness of A2-RGC subtype to high epiretinal electrical stimulation frequency^8^ using our morphologically and biophysically realistic A2 cell model. We modified the A2-cell morphology to divide the cell size into small and large based on both soma and dendritic field sizes. The small cell has soma and dendritic field diameters of 17 µm and 320 µm, respectively and the large cell has soma and dendritic field diameters of 26 µm and 500 µm, respectively.

**Figure 6:**
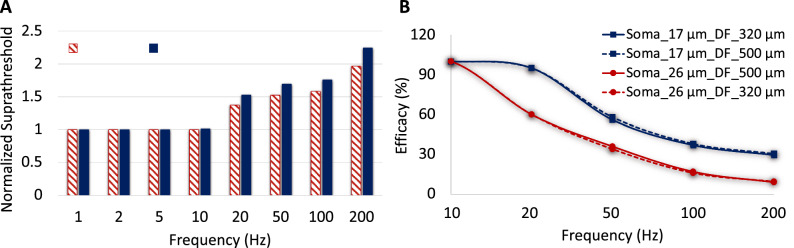
The model verification with in-vitro experimental results from^8^. (**A**) Suprathreshold current required to reach at least 90% efficacy as alterations in stimulus frequency for both small and large A2-cells using an asymmetric cathodic-first stimulus waveform (normalized to 1 Hz). The solid and shaded bars demonstrate the normalized stimulus threshold of large and small cells, respectively. The figure clearly shows the greatest stimulus threshold difference between small and large cells at high frequency. (**B**) Impacts of soma and dendritic field sizes on efficacy for a given pulse amplitude (435 µA cathodic phase amplitude). Small cells are able to maintain their response at higher efficacy compared to large cells.

We applied the same stimulus waveform used for the frequency response of RGCs in^8^, the asymmetric biphasic pulse with a short cathodic phase of 60 µs and 120 µm interphase gap followed with a longer anodic phase of 480 µm duration. Similarly, the efficacy is defined as the minimum current amplitude to achieve more than 90% spikes from the stimulus pulses. Figure 6A shows suprathreshold current normalized to threshold at 1 Hz for both the small and large cells over a range of frequency as defined in^8^. The stimulus threshold remains unchanged up to 10 Hz as expected since the membrane voltage settles back at resting potential prior to the following stimulus pulse. Increasing stimulus frequency increases suprathreshold current required to maintain the same efficacy, and the level of increase in current is higher for large A2-cells at high frequency (Fig. 6A). This indicates the highest threshold percentage difference between low and high frequencies for large cells, which directly affects the probability of generating spikes at high frequency.

There are electrophysiological and morphological factors affecting the responsiveness of RGCs at high stimulus frequency. In this section, similar to the performed experiment, we analyzed effects of cell size on maintaining excitability of cells at high rate of stimulation. Figure 6B demonstrates changes in efficacy as alterations in stimulus frequency. Efficacy reduces as frequency is increased, and large cells have lower efficacy at high repetitive stimulus pulses compared to small cells. This agrees with experiments on A2-RGCs, showing smaller cells can better sustain high stimulation frequency^8^. Using our computational platform, we were able to consider the impact of dendritic field size separately from the soma size. We changed the size of the dendritic field for the given soma diameters of the small and large RGCs (17 µm and 26 µm), comparing solid and dash lines in Fig. 6B. Alterations in the dendritic field size have a negligible effect on the efficacy of both small and large A2 RGCs, which explains the dominant effect of soma size on the excitability of these cells.

### Clinical testing in a patient with retinal implant

**Figure 7:**
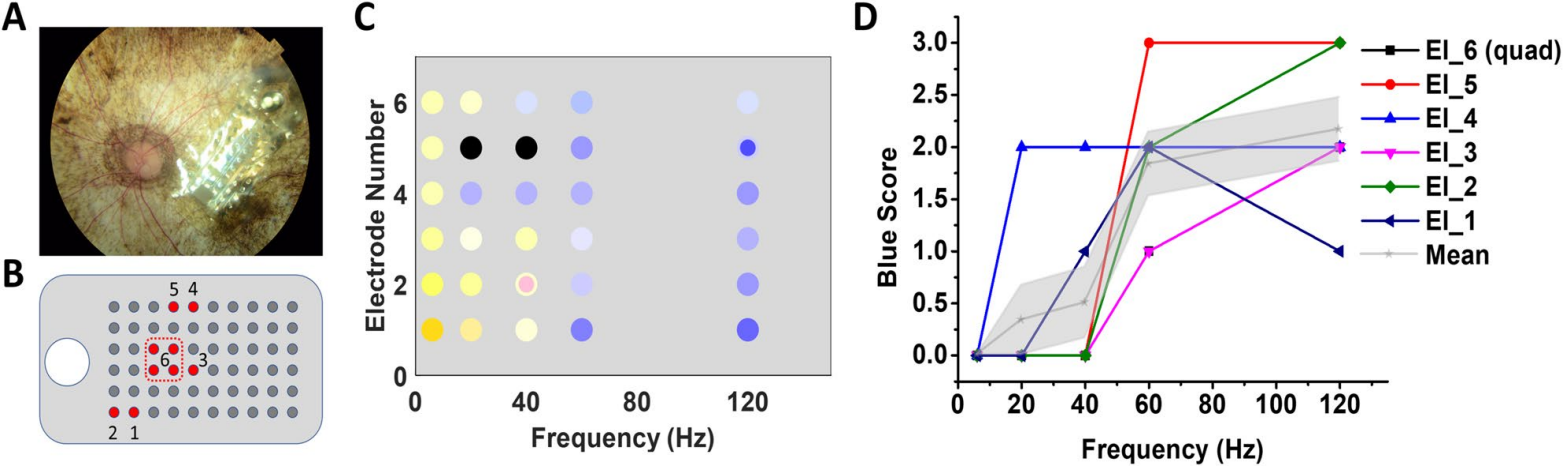
Color perception in a blind RP patient fitted with the Argus II retinal prosthesis. (**A**) Fundus image showing the location of the electrode array on the retina; (**B**) Mapping of the electrodes selected for testing in the visual field; (**C**) Color sensations elicited by different electrodes under frequency modulation; (**D**) Blue scores of the color sensations calculated by the following scaling system: 0—no blue or purple perception; 1—blue or purple sensation reported, but the color is highly unsaturated (saturation ≤ 0.2); 2—more significant blue or purple sensation reported (0.2 < saturation ≤ 0.5); 3—strong blue or purple sensation reported (saturation > 0.5). The gray shaded area represents the standard error.

Earlier studies in blind RP subjects fitted with the Argus II and the IMI retinal prosthesis demonstrated that color sensation could be elicited by electrical stimulation of the photoreceptor-less retina and that the colors perceived may be shifted by the stimulation frequency^32,33^. More recently, Yue et al. found that when phosphene brightness was maintained, increased stimulation frequency consistently shifted phosphene perception to blue tinted colors in 5/7 Argus II subjects tested^32^. These subjects were visually deprived by RP since adolescence or early-to-middle adulthood, having been blind for decades without light or color perception^32^. An example of the changes in color perception in one subject is shown in Fig. 7. The electrode array was implanted in the parafoveal locations superior temporal to the optic disc (Fig. 7A). Color perception was tested in five individual electrodes (Electrodes 1–5) and one group of 4 neighboring electrodes (Electrode Quad 6). Relative locations of these electrodes in the visual field are mapped in Panel B. Hue and saturation of the colors reported were depicted in Panel C, in which two colors simultaneously perceived in one phosphene were presented in concentric rings. When the stimulation frequency increased from 6 to 120 Hz, the phosphene perceived changed from yellow/white dominated colors to blue dominated colors. Other colors such as black and pink were sporadically reported only. Quantification of the blue sensation yielded a blue score that consistently increased with the frequency, suggesting the possibility of using frequency modulation to selectively activate different color pathways in the inner retina, bypassing the cone photoreceptors.

## Discussion

A multi-scale computational study using a combined AM-NEURON model was conducted to further our understanding of the cellular, and potentially color, selectivity of RGC subtypes in the electrically stimulated degenerated retina. We first developed realistic models of the two classified ganglion cells known as D1-bistratified and A2-monostratified. Their responses to electrical stimulation with alterations in stimulation frequency were further evaluated. Our findings show that: (i) the greatest differential firing rate between D1-RGCs and A2-RGCs can be achieved at high stimulation frequency; (ii) with the proper choice of current amplitude at high frequency, D1-RGCs can be selectively activated; (iii) there are electrophysiological and morphological factors influencing RGCs response to high stimulus frequency; (iv) RGCs with a relatively small soma size are more responsive to high stimulus frequency.

Our results show that D1 cells can be selectively activated at a 20 Hz firing rate, similar to the typical stimulus frequency employed in the epiretinal prosthetic systems, which is found to induce stable phosphenes^41,42^ with a proper selection of current amplitude at 200 Hz stimulus frequency. We found that the greatest difference in the current amplitude required to reach 20 Hz firing rate in both cells (~ 22 µA) can be achieved using a high frequency of 200 Hz. The differential current threshold between low and high stimulation frequencies is further supported in Fig. 6A, comparing the suprathreshold current required to reach 90% efficacy (spike probability) for small and large A2 cells using the optimized waveform in^8^. Using our computational platform, we found a small difference in the required current for gaining 90% efficacy between the cells at low stimulation frequency compared to a significant difference in the current amplitude at high frequency, as shown in Fig. 6A (12.5 µA at 10 Hz, compare to 109 µA at 200 Hz). Again, this manifests the greatest chance for selective activation of small cells at high frequency.

We gained additional insights into the underlying mechanisms leading to increased responsiveness of D1 cells and the potential for selective excitation of this cell type at high frequency. We found the greatest impact on the capability of these cells to elicit spikes at high frequency to be related to the soma diameter compared to the axon diameter (Table 1). Our analysis shows that D1 RGCs with in average smaller soma size are better able to follow high repetitive stimulus pulses. We further considered the impact of possible differences in AIS properties between the two RGC subtypes on their response to high frequency stimuli. Recently, a positive correlation has been reported between the length of the AIS and the soma size across a population of α S RGCs^40^. In addition, increase in the length of the SOCB has shown to reduce the stimulation threshold of RGCs to electrical stimulation^38^. Our computational models allowed us to separately investigate the role of modulations in the length of the AH and SOCB on the sensitivity of RGCs to high stimulation frequency. Although reducing the length of SOCB in D1 cells with smaller soma size decreased the responsiveness of this cell to high stimulus frequency, the contribution of soma diameter changes is found to be more significant. Comparing the response of the two cells with simultaneous modulations in the soma diameter, axon diameter, and SOCB length established higher responsiveness of D1 cells compared to A2 cells at a high stimulus frequency (Fig. 5). This finding is consistent with the experiments on frequency response of A2-RGC type, showing that small cells can better sustain high rate of spikes at high frequency^8^.

Consistent with a recent study in α RGCs^39^, we found negligible influence of the AH length and dendritic field size, and a relatively strong impact of soma size on soma RGCs threshold. The significant contribution of the AIS length relative to other morphological factors to the AIS threshold of RGCs with a point-source electrical stimulation was reported in^39^. In our recently published study^12^, an almost two-fold increase in the differential AIS threshold of these morphologically- and biophysically-distinct RGCs using a disk electrode was found for fixed AIS properties of the cells. This indicates the increased sensitivity of RGCs threshold to soma diameter changes using the current large disk electrode of Argus II prosthetic systems, rather than a point source. In the present study, we further explored the enhanced current window required for selective activation of RGCs in response to high stimulus frequency relative to single stimulus pulse and low stimulation frequency. The differences in the biophysical properties, spike width, spike latency, and the duration of the refractory period across RGCs can contribute to the slow-moving firing rate of the A2 cell with increases in the current amplitude, suggesting the reduced potential for preferential excitation of this cell type at high frequency.

It is also worth noting that while there is a positive correlation between soma diameter and axon diameter of monostratified cells, this correlation is shown to be not significant in bistratified RGCs in the primate retina^26^. Therefore, not only smaller soma size, but also relatively larger axon diameter of D1-type would lead to higher chance of spikes in this RGC type at high stimulation frequency. Hence, a plausible explanation behind the domination of the blue percept at high frequency in Argus II patients could be relatively large axon and small soma diameters of small bistratified RGCs, assuming their contributions to “blue-yellow” color opponent pathway in the retinal circuitry^28–31^.

**Figure 8:**
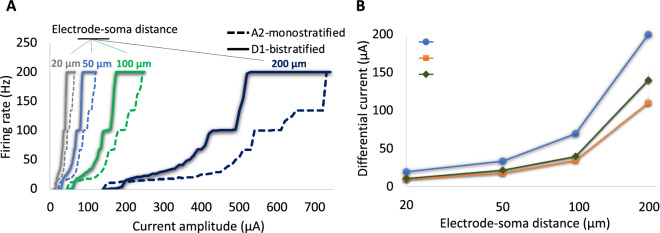
The influence of electrode-cell distance on response and selective activation of RGCs at 200 Hz. (**A**) Firing rates of the A2 and D1 RGCs as a function of current amplitude for four difference electrode-soma distances (20 µm, 50 µm, 100 µm, and 200 µm). (**B**) Current amplitude difference between the two cells required to obtain firing rates (FRs) of 20 Hz, 100 Hz, and 200 Hz with increase in the electrode-soma distance. Data show that the differential firing rate and current amplitude of RGCs increased with increasing electrode-cell distance, suggesting the enhanced chance for preferential activation of D1 cells.

Studies reported the gradual changes in the electrode impedance and therefore the perceptual threshold of Argus I and II implants^43,44^. Increase in electrode to retina distance was shown to increase the perceptual threshold of Argus I subjects^43^. However, the recent clinical data from one subject with Argus II implant reported no significant changes in the electrode-retina distance up to 40 months after implantation, suggesting the contribution of other factors, such as changes in impedance due to electrochemical reactions on the electrode surface, to the perceptual threshold changes of the subject^44^. More tests need to be done clinically to measure the electrode-retina distance/orientation variations across the electrodes and Argus II subjects, and analyze the impact on the perceptual threshold. Using our multi-scale computational modeling platform, we explored the influence of modulations in the electrode-to-retina distance on the response of RGCs at high frequency. So far, we have only considered the response of the two cells for a given 50 µm electrode-to-cell distance. Figure 8A compares the firing rates of the A2 and D1 RGCs as alterations in the current amplitude for 20 µm, 50 µm, 100 µm, and 200 µm electrode-soma distances at 200 Hz. Increased distance between the electrode and cell bodies leads to increased current threshold^9^. Further, we computed the difference in the required current amplitude to reach firing rates of 20 Hz, 100 Hz, and 200 Hz for both cells (Fig. 8B). The differential response of the two cells significantly increases with increase in the electrode-cell distance, suggesting the enhanced chance for selective activation of the D1-bistratified cell.

Recent clinical data reported that 2 of the 7 Argus II subjects did not perceive blue color at high stimulation frequency^32^. Interestingly, the perceptual thresholds in the two non-blue-sensing subjects were found to be lower in average compared to the other 5 subjects. The lowest perceptual threshold was also perceived by the non-blue-sensing subject^32^. Although no significant difference in the electrode-retina interface among the subjects was observed, our computational analysis suggests that even small variations in the electrode-retina distance across the electrodes and among the Argus II subjects can provide a plausible explanation of the blue perception difference between the two groups. The low threshold of the non-blue-sensing subject may indicate the closer electrode-to-retina distance and therefore less likelihood of selectively activating the small bistratified RGCs. Further investigation is required to better understand the correlation between electrode-retina distance and blue sensation of the subjects.

Our computational findings, along with the experimental verifications, suggest that there are electrical stimulation parameters with the greatest contribution to changes in RGCs stimulus threshold and perceptual threshold. These parameters consist of stimulation frequency, electrode-retina distance, electrode impedance, and pulse duration that can play significant roles in possible selective activation of different RGCs, as well as avoiding activation of RGCs axon bundles. For example, short stimulus pulse durations with relatively higher stimulation thresholds have proven effective in achieving a more focal response in RGCs^12–15^. While the impact of pulse width modulations on the blue sensation of the Argus II subjects was found to be negligible^32^, we will further investigate the influence of pulse duration changes on selective activation of RGCs.

Increasing both stimulation frequency and current amplitude results in an increase in phosphene brightness with a more pronounced impact of stimulation frequency^45^. Saturation in brightness and increase in phosphene size have been reported with increasing current amplitude^45,46^. Lowering the current amplitude as the simulation frequency increases is required for controlling the perceptual brightness and perceiving the color of the phosphene, including the blue percept by Argus II subjects^32^. Our computational results indicate that increasing either the stimulation frequency or current amplitude leads to an increase in firing rate. For example, as shown in Fig. 6B, for a given current amplitude of 435 µA, while the efficacy of small cells at 200 Hz is 30.5% (0.305 × 200 = 61 Hz firing rate), the efficacy is 100% at 20 Hz which means the firing rate of 20 Hz. Taken together, evidence suggests a positive correlation between rate of RGCs spikes and phosphene brightness. Therefore, electrical stimulation at high frequency with a proper current amplitude tuning for brightness control results in a better chance for selective activation of RGCs and sensation of blue percept in the subjects.

The current amplitudes associated with the maximum firing rates of A2 and D1 RGCs do not necessarily mean the saturation of perceptual brightness in the subjects. While the maximum spiking frequency of RGCs has been reached with direct activation, network-mediated response of RGCs may further alter the firing rates of RGCs. We did not consider the presynaptically driven response of RGCs in the present study. Further, high frequency of stimulation may result in phosphenes fading and cessation of indirect RGCs excitation^47,48^. The cross-talk across the electrodes using synchronous stimulus pulses has been reported to increase the brightness of phosphenes as well^49^. Given direct and indirect activations of RGCs using the 0.46 ms pulse width, the rate of RGCs spikes leading to a moderate perceptual brightness of the subjects is not known. Therefore, we compared the differential amplitude of RGCs leading to 20 Hz, 100 Hz, and 200 Hz firing rates of the cells at 200 Hz frequency stimuli as depicted in Fig. 8B.

Center-surround receptive field structure (S ON versus L + M OFF, S: short L: long, M: middle wavelength), or “blue-yellow” opponent visual pathway has already been identified. Short wavelength sensitive (S) cone photoreceptors make selective connection with S-cone ON bipolar cells, and L and M cones are presynaptic to OFF cone bipolar cells, then signals from these pathways are transmitted to inner and outer dendrites of small bistratified ganglion cells^28–31,50^. A recent study has suggested that, although small bistratified RGCs play a role in blue-yellow perception in periphery, this percept is mediated by other pathways in central retina^51^. This hypothesis is based on testing in patients with congenital stationary night blindness (CSNB), who lack the metabotropic glutamate receptor (mGluR6), which is leading to loss of response sensitivity to ON pathway and presumably eliminating the synaptic connection from S cone to S cone ON bipolar cells^52^. Terasaki et al.^52^ observed that blue/yellow color vision of these subjects was intact in central retina, but impaired in peripheral retina, suggesting S-ON bipolar cells and therefore small bistratified RGCs do not contribute to blue-yellow perception in central retina. However, most recently Thoreson and Dacey^53^ have stated that while S-ON response is diminished in CSNB patients, L + M OFF response remains preserved (see Fig. 10A in^53^),^54^, and OFF inputs can be sufficiently strong enough to carry information about the light response and compensate for lack of inputs from ON pathway. They further raised this theory to be doubtful by stating: “there are also no obvious deficits in the perception of ON versus OFF luminance contrast in CSNB patients”^53^.

In the primate visual system, there are three pathways: parvocellular (P), magnocellular (M), and koniocellular (K)^55,56^. Parasol ganglion cells with large soma and dendritic field size project to the M pathway and are color insensitive^57^. However, midget and bistratified ganglion cells with small cell bodies and dendritic fields send neural signals to the P and K pathways and they are involved in color vision^58–60^. Even if small bistratified RGCs are not involved in blue-yellow color opponency, the large soma size of color insensitive parasol cells and small soma size of color selective midget cells possibly explain the importance of our computational findings, particularly due to the fact that the subjects could occasionally see other colors such as purple and gold at high frequency as well^32^.

Our results are limited to only two types of RGCs and the sensitivity of other RGCs to high stimulation frequency requires further investigation. Since the band information is not clearly identified for the two RGCs, in this work we assumed identical axonal biophysics for both cells and focused on morphological factors such as soma, dendritic field, axon diameters, and the length of the AIS. The impact of retinal degeneration on changes in the morphometric parameters of the cells assumed to be negligible in the present study. We identified the soma diameter, SOCB length, and biophysical differences between the cells as critical factors affecting responsiveness of RGCs at high frequency. Future studies will incorporate morphologically and electrophysiologically other types of RGCs with a wide range of cell body sizes as well as the effect of electrode position on response of RGCs to high stimulation frequency. We will develop a synthetic retinal network, modeling a large population of different RGCs and analyzing the sensitivity of cells response to various morphological changes and modulations in electrode orientations with respect to the surface of the retina. We will further design electrical stimulation waveforms with the aim of independent activation of various RGCs at high stimulation frequency.

This study is motivated by our intent to identify mechanisms that will allow us to potentially encode additional information such as color in a visual prosthetic system. Our multi-scale computational framework helped us further our understanding of the color-coding sensitivity in the electrically stimulated degenerated retina. Assuming significant contribution of small bistratified retinal ganglion cells in blue-yellow color vision, we were able to selectively target these cells with in average small soma size at high stimulus frequency with a careful modulation of current amplitude. Our computational finding may be correlated with the clinical study in patients with epiretinal prostheses showing that stimulation frequency played a role in the percept of colors, and particularly the blue percept at high frequency. The verification of the computational models with the experimental data in rats and preliminary experimental results in patients with epiretinal implants, allowed us to better elucidate the underlying mechanisms of differential percept and provide more insights toward the development of visual prosthetic systems with increased information content for the patient.

## Methods

### Admittance method/NEURON computational framework

In this work, we utilized our three-dimensional Admittance Method (AM)/NEURON multi-scale computational modeling platform^12,61–73^ to predict the electric fields generated inside retinal tissue, coupled to multi-compartmental models of neurons in order to determine the activation of realistic RGCs. The Admittance Method linked with NEURON has proven a powerful approach not only for studies of field distribution inside the tissue due to electrical stimulation, but also providing a platform to analyze realistic representations of various cell types^12,61–73^.

### Admittance method: constructing the retina tissue and electrodes

In this approach, computational models of the retina tissue and implant electronics are created through discretization of segmented images, and electrical properties are assigned to each voxel of the model. Current sources are applied as input and the resulting voltages are computed at each node. A linear interpolation function is used to obtain the voltage at the center of each neuronal compartment, which is utilized for the computation of the neural response using the NEURON simulator (v7.4; https://neuron.yale.edu/neuron)^74^. Further details can be found in^61–67^. The AM-NEURON computational platform has been recently parallelized by our group and accommodates adaptive multiresolution meshing. In this work, the minimum model resolution was set to 10 μm and we merged at most 64 voxels in areas of lower resolution, away from boundaries between tissues.

To represent the degenerated retina tissue, the thickness of the outer part of the retina, which consists of outer plexiform and outer nuclear layers, were mostly reduced in size. The retina laminar properties are identical to those utilized in our previous work^62^. The computational model of a stimulating electrode of diameter 200 μm is placed on the top-center of the bulk retina tissue, which is discretized in 2 million computational cells, and is positioned 50 µm from the cell bodies of computational models of the RGCs unless otherwise stated. The resistivity of platinum (10.6 × 10^–8^ Ω m) is utilized in the model of the electrode, which is surrounded by insulating material. The admittance method was then used to solve the voltage generated inside the tissue by the stimulus current. Unless otherwise specified, we used a symmetric charge-balanced biphasic pulse of constant pulse width (0.5 ms) with no interphase gap (IPG), and amplitude modulations from 20 µA to 140 µA. The stimulation frequencies ranging from 6 to 200 Hz were considered. The parameters used are identical to those used in the experimental studies of the patients with epiretinal implants^32^. Resulting extracellular voltages were applied to multi-compartment models of neurons and computation executed using embedded NEURON software. Neuronal responses of individual retinal ganglion cells were then recorded.

### NEURON: retinal ganglion cell models

The morphology of ganglion cell types was extracted as SWC files from the NeuroMorpho dataset^75,76^ and imported to NEURON software^74^ as shown in Fig. 1. The extracted cells are of types A2 and D1, and their morphological parameters can be found in^77^. These parameters are provided in Fig. 1 and utilized for our AM-NERUON simulations unless otherwise noted. D1-bistratified cells consist of two levels of dendritification, in which one layer of the dendritic tree is ramified inside the inner part, and another is in the outer section of the inner plexiform layer. The dendritic structure of the A2-monostratified cell types is only distributed in the inner part of the inner plexiform layer.

These morphologically realistic cells are compartmentalized and their responses to electrical stimulation are solved based on multi-compartment Hodgkin–Huxley models. Each compartment includes several ionic channels, and they are modeled as voltage-dependent conductances in parallel with the membrane capacitance. In addition to the five ionic channel models from Fohlmeister and Miller^78,79^ for the ganglion cells, two more ionic currents have been considered to more accurately represent the intrinsic electrophysiological properties of different ganglion cell types including the difference between ON and OFF cell types and the phenomenon of rebound excitation, which plays a fundamental role in encoding visual percepts^80^. The hyperpolarization-activated, and the low voltage activated (LVA) calcium ionic channels were modelled as in^81,82^ respectively. More details can be found in^37,61,80^. The expressions of rate constants for different ionic channels are given in Supplementary Table S1.

Recently, a single-compartment model of ganglion cell was used to find the constraints for the maximum ionic conductance values, in which the model output can replicate the electrophysiological properties of different RGC types^37^. We first reproduced the results in^37^ and then further developed the RGC models to include multi-compartmental representations and tuned the density of ion channels accordingly in soma, dendrites and axon. In addition, since the axons were missing from the available morphologies, we extracted them from another dataset, modified to include the axon initial segment, and patched them to the cell body of both cells. The morphological properties of the axon are adapted from^38^ as shown in Fig. 1. The experimentally recorded signals of A2 and D1 cells in^37^ were used for model tuning. The range of variation in the density of ion channels of the dendrites, and axon is based on the constraints demonstrated by Fohlmeister et al.^79^. The tuned biophysical properties of both A2 and D1 cells for the soma, dendrites, and axon are provided in Supplementary Table S2 and S3. Supplementary Fig. S1 shows that the morphologically and biophysically realistic models of RGCs closely reproduce the measured electrophysiological responses provided in^37^. For this validation, intracellular hyperpolarizing step currents of 200 pA with 400 ms duration were injected to the cells and their responses were recorded from the cell body (soma) running NEURON simulations. As illustrated, the RGC’s model can closely replicate the behavior of the experimentally recorded cells, including the rebound excitation phenomenon, which is described as action potentials initiation after termination of a hyperpolarizing current.

### Admittance method linked with NEURON

The multi-compartment models of neurons in the simulation platform are integrated in our computational multiscale simulation package. For the extracellular stimulation of the retina tissue, the Admittance Method was used to calculate the resulting voltage at each node for a given input current. The voltage at the center of each voxel was estimated using a linear interpolation function. Since the Admittance Method and NEURON use the same coordinates, a computational code was developed to superimpose the potential computed in the tissue volume into the NEURON model and apply it as an extracellular voltage, using the “extracellular” mechanism built into NEURON software, to each compartment in the Hodgkin–Huxley circuit in series with the membrane^12,61–71^.

Individual responses of both A2 and D1 ganglion cell types to extracellular epiretinal stimulation were computed using a range of stimulus frequency with the goal of identifying the responsiveness of RGCs at high frequency of stimulation^61^. This will further help translate from biophysically realistic models of the retinal ganglion cells to how color percepts might be elicited from patients with retinal prostheses.

## Supplementary Information

Supplementary Information.
