# Publisher Correction: Color and cellular selectivity of retinal ganglion cell subtypes through frequency modulation of electrical stimulation

**DOI:** 10.1038/s41598-021-92050-0

**Published:** 2021-06-14

**Authors:** Javad Paknahad, Kyle Loizos, Lan Yue, Mark S. Humayun, Gianluca Lazzi

**Affiliations:** 1 Department of Electrical Engineering, University of Southern California, Los Angeles, CA USA; 2 The Institute for Technology and Medical Systems (ITEMS), Keck School of Medicine, University of Southern California, Los Angeles, CA USA; 3 Roski Eye Institute, University of Southern California, Los Angeles, CA USA; 4 Departments of Ophthalmology and Biomedical Engineering, University of Southern California, Los Angeles, CA USA

Correction to: *Scientific Reports* https://doi.org/10.1038/s41598-021-84437-w, Published online 04 March 2021

The original version of this Article contained an error in Figure 1, where the lower coordinate system was left-handed, while the upper coordinate system was right-handed.

Furthermore, the labels in Figure 6A and Figure 8B were omitted.

The original Figures 1, 6 and 8 and accompanying legends appear below.

**Figure 1:**
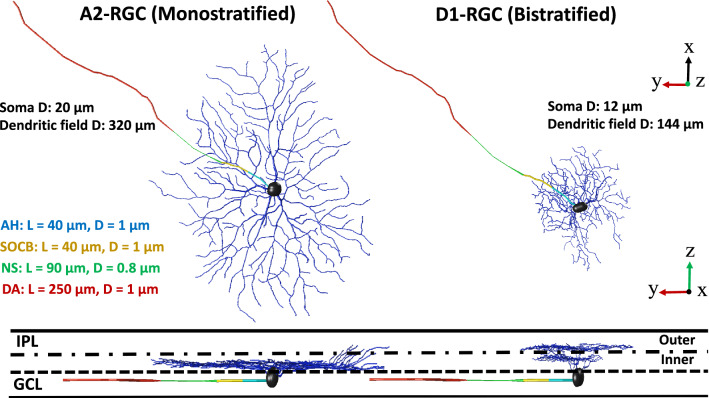
A2 and D1 realistic morphologies as implemented and coded in our multiscale Admittance Method/NEURON computational platform^61–73^. Left: A2-monostratified RGC ramified in the inner part of inner plexiform layer and has a larger soma and dendritic field diameters. Right: D1-bistratified, their dendrites are placed in both inner and outer part of the inner plexiform layer and this cell has relatively smaller soma and dendritic field diameters. GCL: ganglion cell layer; IPL: inner plexiform layer; AH: axon hillock; SOCB: sodium channel band; NS: narrow segment; DA: distal axon; L: length of each band; D: diameter. The morphology of RGCs was extracted from the NeuroMorpho dataset^75–77^.

**Figure 6:**
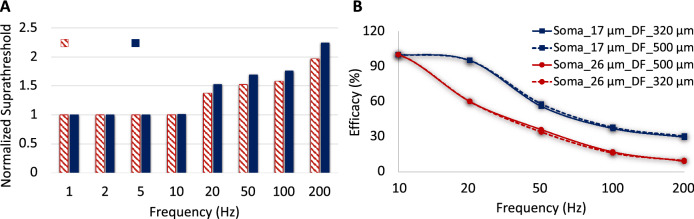
The model verification with in-vitro experimental results from^8^. (**A**) Suprathreshold current required to reach at least 90% efficacy as alterations in stimulus frequency for both small and large A2-cells using an asymmetric cathodic-first stimulus waveform (normalized to 1 Hz). The solid and shaded bars demonstrate the normalized stimulus threshold of large and small cells, respectively. The figure clearly shows the greatest stimulus threshold difference between small and large cells at high frequency. (**B**) Impacts of soma and dendritic field sizes on efficacy for a given pulse amplitude (435 µA cathodic phase amplitude). Small cells are able to maintain their response at higher efficacy compared to large cells.

**Figure 8:**
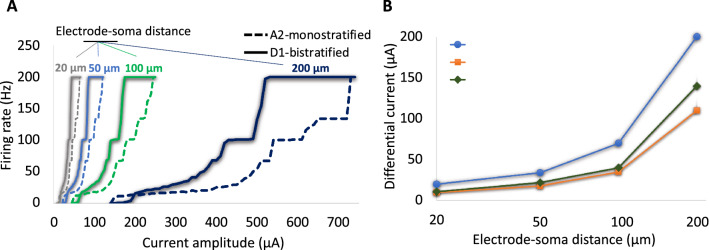
The influence of electrode-cell distance on response and selective activation of RGCs at 200 Hz. (**A**) Firing rates of the A2 and D1 RGCs as a function of current amplitude for four difference electrode-soma distances (20 µm, 50 µm, 100 µm, and 200 µm). (**B**) Current amplitude difference between the two cells required to obtain firing rates (FRs) of 20 Hz, 100 Hz, and 200 Hz with increase in the electrode-soma distance. Data show that the differential firing rate and current amplitude of RGCs increased with increasing electrode-cell distance, suggesting the enhanced chance for preferential activation of D1 cells.

The original Article has been corrected.

