# Supplementary material for: Color and cellular selectivity of retinal ganglion cell subtypes through frequency modulation of electrical stimulation
Source: Sci Rep. 2021 Mar 4;11:5177. doi: 10.1038/s41598-021-84437-w (PMC7933163; doi:10.1038/s41598-021-84437-w)
Supplement: Supplementary file 1 — Supplementary Information. [file 41598_2021_84437_MOESM1_ESM.pdf]

Supplementary Information

Color and Cellular Selectivity of Retinal Ganglion Cell Subtypes Through Frequency Modulation of Electrical Stimulation

Javad Paknahad, Kyle Loizos, Lan Yue, Mark Humayun, and Gianluca Lazzi

SUPPLEMENTARY TABLE S1

RATE CONSTANTS OF IONIC CURRENTS

Na Channel	$\alpha_m = \dfrac{-0.6(E+30)}{e^{-0.1(E+30)}-1}$ $\alpha_h = 0.4\ e^{-(E+50)/20}$	$\beta_m = 20\ e^{-(E+55)/18}$ $\beta_h = \dfrac{6}{e^{-0.1(E+20)}+1}$
Ca Channel	$\alpha_c = \dfrac{-0.3(E+13)}{e^{-0.1(E+13)}-1}$	$\beta_c = 10\ e^{-(E+38)/18}$
K Channel	$\alpha_n = \dfrac{-0.02(E+40)}{e^{-0.1(E+40)}-1}$	$\beta_n = 0.4\ e^{-(E+50)/80}$
A Channel	$\alpha_A = \dfrac{-0.006(E+90)}{e^{-0.1(E+90)}-1}$ $\alpha_{h_A} = 0.04\ e^{-(E+70)/20}$	$\beta_A = 0.1\ e^{-(E+30)/10}$ $\beta_{h_A} = \dfrac{0.6}{e^{-0.1(E+40)}+1}$
h Channel	$\alpha_l = e^{0.08316(E+75)}$	$\beta_l = e^{0.033264(E+75)}$
T Channel	$\alpha_{m_T} = \left(1.7 + e^{-(E+28.8)/13.5}\right)^{-1}$ $\alpha_{h_T} = e^{-(E+160.3)/17.8}$ $\alpha_d = \dfrac{1+e^{(E+37.4)/30}}{240\left(0.5+\left(0.25+e^{(E+83.5)/6.3}\right)^{0.5}\right)}$	$\beta_{m_T} = \dfrac{e^{-(E+63)/7.8}}{1.7+e^{-(E+28.8)/13.5}}$ $\beta_{h_T} = \alpha_{h_T}((0.25+$ $e^{(E+83.5)/6.3}\big)^{0.5} - 0.5\alpha_{h_T}$ $\beta_d = \alpha_d\left(0.25+e^{(E+83.5)/6.3}\right)^{0.5}$

SUPPLEMENTARY TABLE S2

MAXIMUM IONIC CONDUCTANCE VALUES FOR A2 AND D1 CELLS [S/CM^2].

	RGC types			
	A2		D1	
	**Soma**	**Dendrite**	**Soma**	**Dendrite**
g_{Na}	0.3	0.1	0.2	0.08
g_K	0.12	0.05	0.211	0.08
$g_{K,A}$	3*g_K	3*g_K	3*g_K	3*g_K
$g_{K,Ca}$	0.004*g_K	0.004*g_K	0.004*g_K	0.004*g_K
g_{Ca}	0.137	0.05	0.013	0.01
g_h	0	0	0.0001	3e-5
g_T	0.004	0	0.002	0.001

SUPPLEMENTARY TABLE S3

MAXIMUM IONIC CONDUCTANCE VALUES OF THE AXON FOR A2 AND D1 CELLS [S/CM^2].

	AH	SOCB	NS	DA
g_{Na}	0.8	2.4	0.9	0.8
g_K	0.6	0.8	0.6	0.6
$g_{K,A}$	3*gK	3*gK	3*gK	3*gK

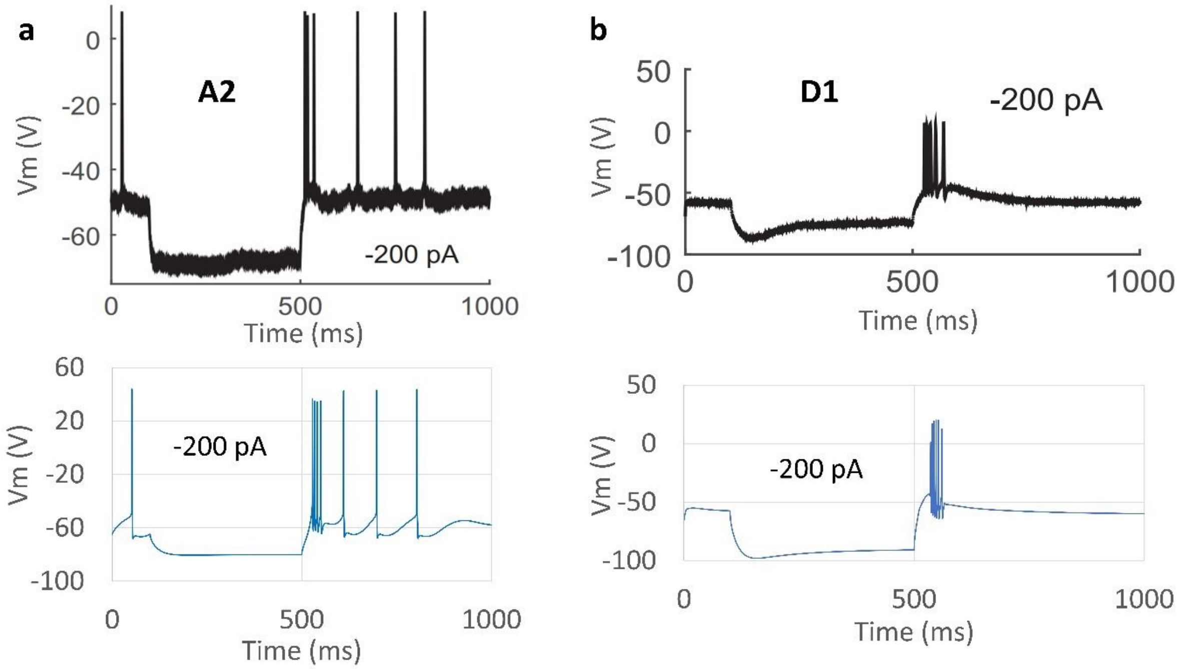

Supplementary Fig. S1. Comparison between experimental (top) and computational (bottom) membrane voltages in the cell body (soma) in response to intracellular stimulation. The hyperpolarizing step current stimulation was applied between 100 ms and 500 ms. (**a**): A2 cell; (**b**): D1 cell. Experimental data obtained from [37].
